# Developmental Neurotoxicity Screen of Psychedelics and Other Drugs of Abuse in Larval Zebrafish (*Danio rerio*)

**DOI:** 10.1021/acschemneuro.2c00642

**Published:** 2023-02-08

**Authors:** Robert J. Tombari, Paige C. Mundy, Kelly M. Morales, Lee E. Dunlap, David E. Olson, Pamela J. Lein

**Affiliations:** † Department of Chemistry, University of California, Davis, Davis, California 95616, United States; ‡ Department of Molecular Biosciences, University of California, Davis, Davis, California 95616, United States; § Department of Biochemistry & Molecular Medicine, School of Medicine, University of California, Davis, Sacramento, California 95817, United States; ∥ Center for Neuroscience, University of California, Davis, Davis, California 95618, United States; ⊥ Institute for Psychedelics and Neurotherapeutics, University of California, Davis, Davis, California 95616, United States

**Keywords:** Behavioral abnormalities, hallucinogens, psychoplastogens, psychostimulants, teratology

## Abstract

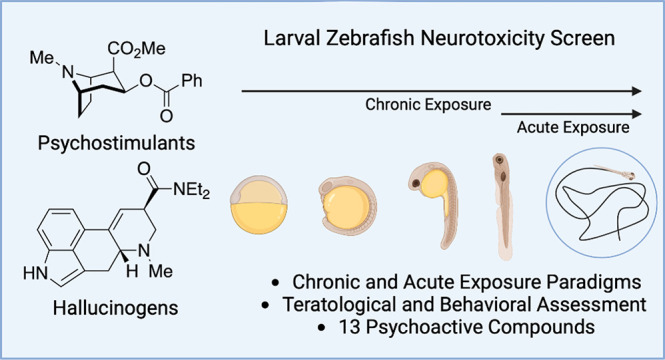

In recent years, psychedelics have garnered significant interest as therapeutic agents for treating diverse neuropsychiatric disorders. However, the potential for these compounds to produce developmental neurotoxicity has not been rigorously assessed, and much of the available safety data is based on epidemiological studies with limited experimental testing in laboratory animal models. Moreover, the experimental safety data available thus far have focused on adult organisms, and the few studies conducted using developing organisms have tested a limited number of compounds, precluding direct comparisons between various chemical scaffolds. In the present study, 13 psychoactive compounds of different chemical or pharmacological classes were screened in a larval zebrafish model for teratological and behavioral abnormalities following acute and chronic developmental exposures. We found that the psychedelic tryptamines and ketamine were less neurotoxic to larval zebrafish than LSD and psychostimulants. Our work, which leverages the advantage of using zebrafish for higher throughput toxicity screening, provides a robust reference database for comparing the neurotoxicity profiles of novel psychedelics currently under development for therapeutic applications.

## 1 Introduction

Classic psychedelics (e.g., lysergic acid diethylamide, LSD; *N,N*-dimethyltryptamine, DMT; and psilocybin, PSY) and related compounds (e.g., 3,4-methylenedioxymethamphetamine, MDMA and ibogaine, IBO) are mind-altering drugs often used for religious or recreational purposes.^1^ However, over the past decade, a resurgence of clinical research on these compounds has shifted perceptions of psychedelics as neurotoxic substances to potentially beneficial therapeutics for medically intractable psychiatric conditions.^2^ Emerging clinical evidence suggests that psychedelics can produce rapid and persistent beneficial outcomes in the treatment of depression, anxiety, post-traumatic stress disorder (PTSD), and substance use disorder.^3,4^ Studies by our group and others suggest that ketamine (KET), MDMA, and classic psychedelics elicit their therapeutic effects by rapidly promoting neuronal plasticity in adult prefrontal cortical neurons,^5−7^ leading to their classification as psychoplastogens.^8^

The use of psychedelics for recreational purposes, their increasing use for therapeutic applications, and the rapid development of new psychoplastogens collectively underscore the need for data regarding the safety profiles of these compounds. Particularly concerning is the paucity of data regarding potential adverse effects of these drugs on early neurodevelopment. Psychedelics, like most psychotropic drugs, have the potential to cross the placenta and can be excreted in breast milk.^9^ Moreover, they can be used by people of childbearing age for both recreational and medicinal purposes.^10^ With respect to the latter, KET is being administered in clinical trials as a potential treatment for postpartum depression.^11^ Extensive evidence indicates that prenatal exposure to the psychostimulants, amphetamine (AMPH), MDMA, methamphetamine (METH), and cocaine (COCN) can adversely affect offspring by slowing intrauterine growth, resulting in low birth weights and deficiencies in early cognitive and motor development.^12−14^ However, there are far less data available regarding the effects of hallucinogenic psychoplastogens on neurodevelopment.

Current methods for assessing developmental neurotoxicity that use rodent models are time-intensive and costly, limiting the number of compounds that can be assessed.^15^ Zebrafish (*Danio rerio*) is a well-established vertebrate model widely used for assessing the neurodevelopmental effects of drugs because they combine the genetic and physiological advantages of mammalian models with the higher throughput capabilities and genetic manipulability of invertebrate models.^16,17^ The fundamental mechanisms of neurodevelopment are highly conserved between zebrafish and humans,^18^ with zebrafish expressing gene orthologs for >70% of human genes, 82% of human disease-causing proteins, and 85% of known human drug targets.^19^ Zebrafish have many of the same sensory modalities as humans and exhibit an extensive behavioral repertoire, including affective and depressive-like behavior.^20^ A key consideration in considering zebrafish as an experimental platform for screening psychedelics for potential developmental neurotoxicity is that they express the same major neuromodulator systems as humans, including serotonin, dopamine, and norepinephrine.^21^

While others have investigated neurodevelopmental effects of select psychedelic compounds, no study currently exists that systematically compares hallucinogenic psychedelics from distinct chemical scaffolds. The main goal of the present study was to compare the relative potential for inducing developmental neurotoxicity across a wide structural array of these compounds. We selected diverse psychoactive compounds that encompassed a variety of chemical scaffolds and pharmacological profiles (Table 1). These include psychostimulants, such as AMPH, 3,4-methylenedioxyamphetamine (MDA), MDMA, METH, and COCN that impact monoamine concentrations in the brain,^22−26^ and hallucinogens from diverse chemical classes, including LSD, 2,5-dimethoxy-4-iodoamphetamine (DOI), DMT, psilocin (PSI), PSY, IBO, KET, and scopolamine (SCOP). Overall, our findings indicate that over half of these psychoactive compounds altered behavior without producing overt teratogenic effects, raising concerns about the safety of these compounds in people who can become pregnant.

**Table 1:**
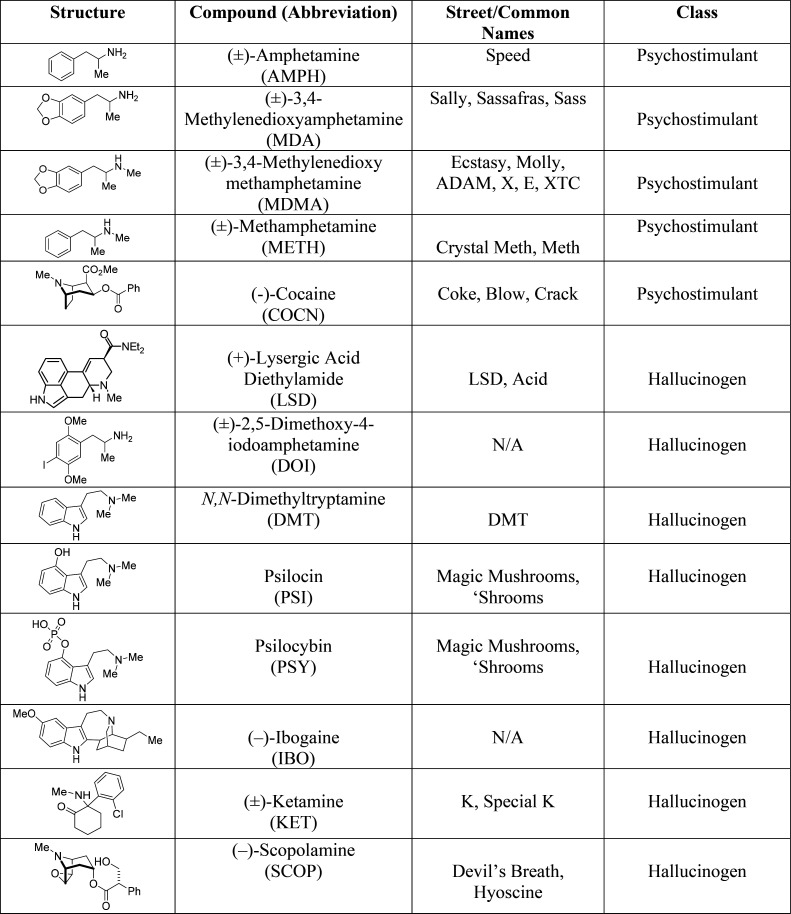
Structures and Pharmacology of Compounds Investigated in this Study

## 2 Results and Discussion

### Teratological and Photomotor Effects of Chronic Exposure During Early Development

**Figure 1:**
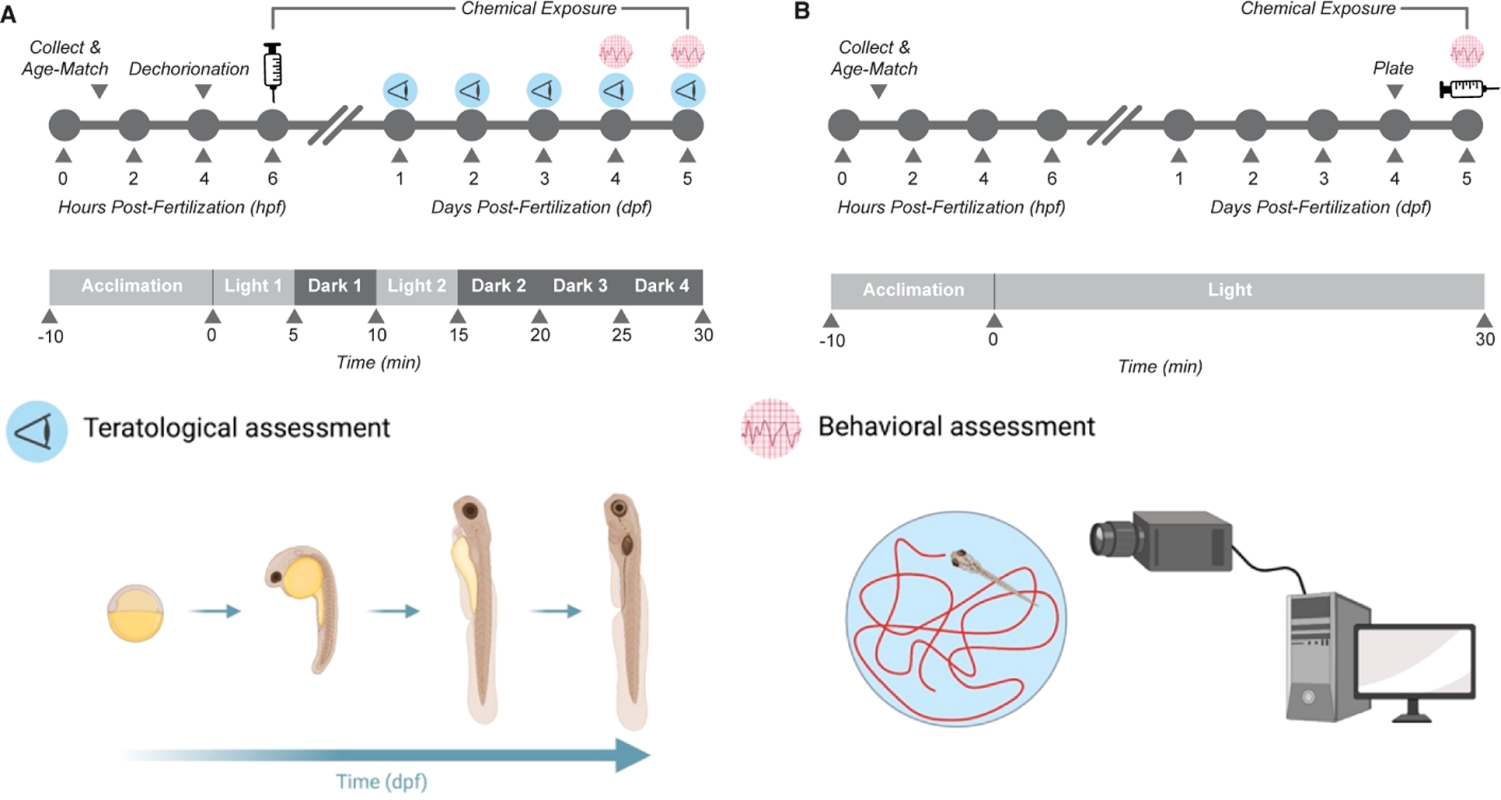
Experimental paradigms for chronic and acute developmental exposures. (A) Chronic exposure: Age-matched embryos were enzymatically treated to remove the chorion at 4 hpf. Dechorionated embryos were transferred into 96-well plates containing embryo medium and exposed to vehicle control (0.1% DMSO) or one of the psychoactive compounds at 6 hpf. All compounds were tested in three replicate experiments. Plates were maintained at 28.5 ± 0.5 °C under a 14 h light/10 h dark cycle until 5 dpf. Exposure solutions were not changed throughout the exposure period. Each day throughout the exposure period, mortality and teratology were assessed, and at 4 and 5 dpf, photomotor behavior was assessed. The behavior assay consisted of a 10 min acclimation period in the light followed sequentially by a 5 min light cycle (Light 1), a 5 min dark cycle (Dark 1), a 5 min light cycle (Light 2), and 3 consecutive 5 min dark cycles (Dark 2, Dark 3, and Dark 4). (B) Acute exposure: Embryos raised in Petri dishes until 4 dpf were transferred into 96-well plates containing embryo medium and exposed to vehicle control (0.1% DMSO) or one of the psychoactive compounds at 5 dpf after a 10 min light phase. Acute behavioral analysis was conducted for a single 30 min light phase immediately following exposure. All compounds were tested in three replicate experiments.

In our initial studies, zebrafish were chronically exposed to varying concentrations of the compounds listed in Table 1 via static waterborne exposure beginning 6 h postfertilization (hpf) and continuing to 5 days postfertilization (dpf).^27^ At 6 hpf, gastrulation has commenced, which is developmentally comparable to a two-week old human embryo. At 10 hpf, the neural plate has formed, and by 30 hpf, heartbeats can be observed.^28^ Zebrafish embryos obtain nutrients from their yolk sacs until 5 dpf, eliminating the need to supply external food sources.^29^ Since zebrafish develop at an accelerated rate relative to humans, exposures from 6 hpf to 5 dpf encompass multiple developmental stages. Thus, by utilizing larval zebrafish, our chronic exposure paradigm captures all the major stages of prenatal and early postnatal human neurodevelopment. Teratological effects were quantified each day of exposure, and photomotor behavior was assessed at 4 and 5 dpf (Figure 1A).^30^

Fish were examined daily for gross teratological effects that included accelerated or delayed midbrain-hindbrain barrier formation (only relevant at 1 dpf), axis malformations (including somite formation, forward or backward bent axis), craniofacial malformations (including eyes, brain, and jaw), caudal and pectoral fin malformations, and edema of the pericardium or yolk sac. At the concentrations tested, none of the compounds screened in this study caused statistically significant teratological effects compared to vehicle control animals (Figure S1). It is important to note that since all exposures were static, there are possible adsorptive losses of compounds to the walls of the polystyrene plates over the exposure period. However, we note that adsorption is primarily an issue for highly hydrophobic compounds such as polycyclic aromatic hydrocarbons,^31^ and is less of a concern when studying compounds that possess a basic amine and are relatively water-soluble. Nonetheless, we cannot definitively rule out the possibility that negative hits (results that were not statistically significant from the vehicle control) are an artifact of chemical partitioning.

Photomotor behavior, which was quantified using several parameters (Table 2), was assessed at 4 and 5 dpf. Sudden changes in light levels between light and dark phases of the assay evoked discrete swimming patterns with fish moving more during dark phases and less during light phases.^32^ An increase in any of the parameters analyzed, but particularly freezing duration, turn angle, angular velocity, mean meander, or total meander, is often associated with anxiogenic or fear/escape responses of the fish.^33^ Conversely, a decrease in overall movement or loss of normal motor function, known as akinesia, which manifests as a decrease in parameters such as mean distance traveled, mean velocity, cruising duration, and bursting duration, can result from exposure to dopamine-depleting drugs.^33^

**Table 2:** Photomotor Behavioral Parameters

Measurement	Unit
Mean Distance	mm
Mean Velocity	mm s^–1^
Cruising Duration	s
Bursting Duration	s
Freezing Duration	s
Turn Angle	Degree (°)
Angular Velocity	° s^–1^
Mean Meander	° mm^–1^
Total Meander	° mm^–1^

**Figure 2:**
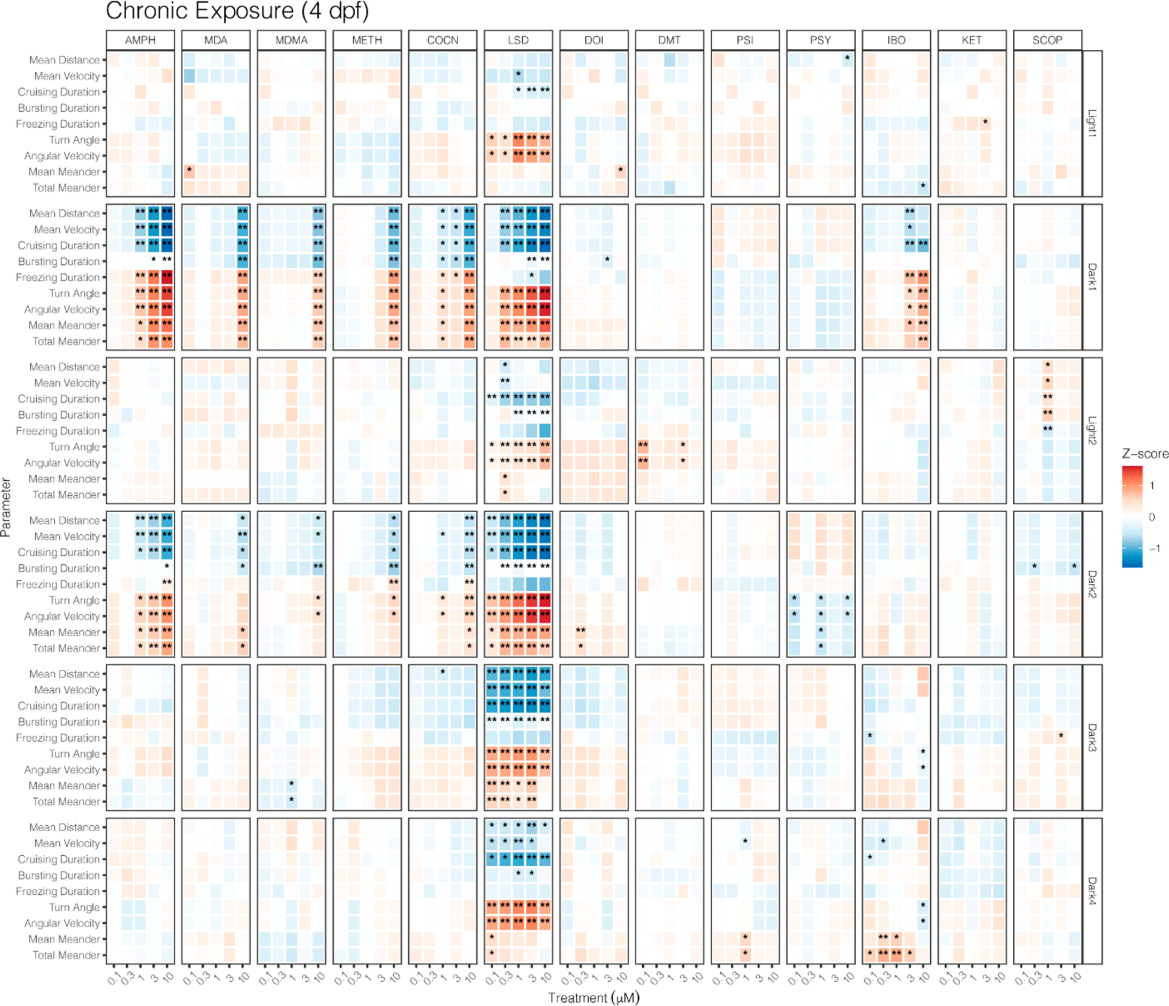
Z-score heatmap and statistical analysis of photomotor behavioral at 4 dpf after chronic developmental exposure. Top *x*-axis: compounds screened at 4 dpf after chronic developmental exposure. Bottom *x*-axis: concentrations of compounds. Left *y*-axis: tracked and analyzed parameters (see Table 2). Right *y*-axis: light cycles (see Figure 1). Z-score heatmap shading indicates increases (red shading) and decreases (blue shading) compared to control fish (Vehicle: 0.1% (v/v) DMSO). Asterisks (*, **) indicate significant differences (*p* < 0.05, *p* < 0.01, respectively) compared to control fish (*n* = 41–48 fish from three separate spawns).

**Figure 3:**
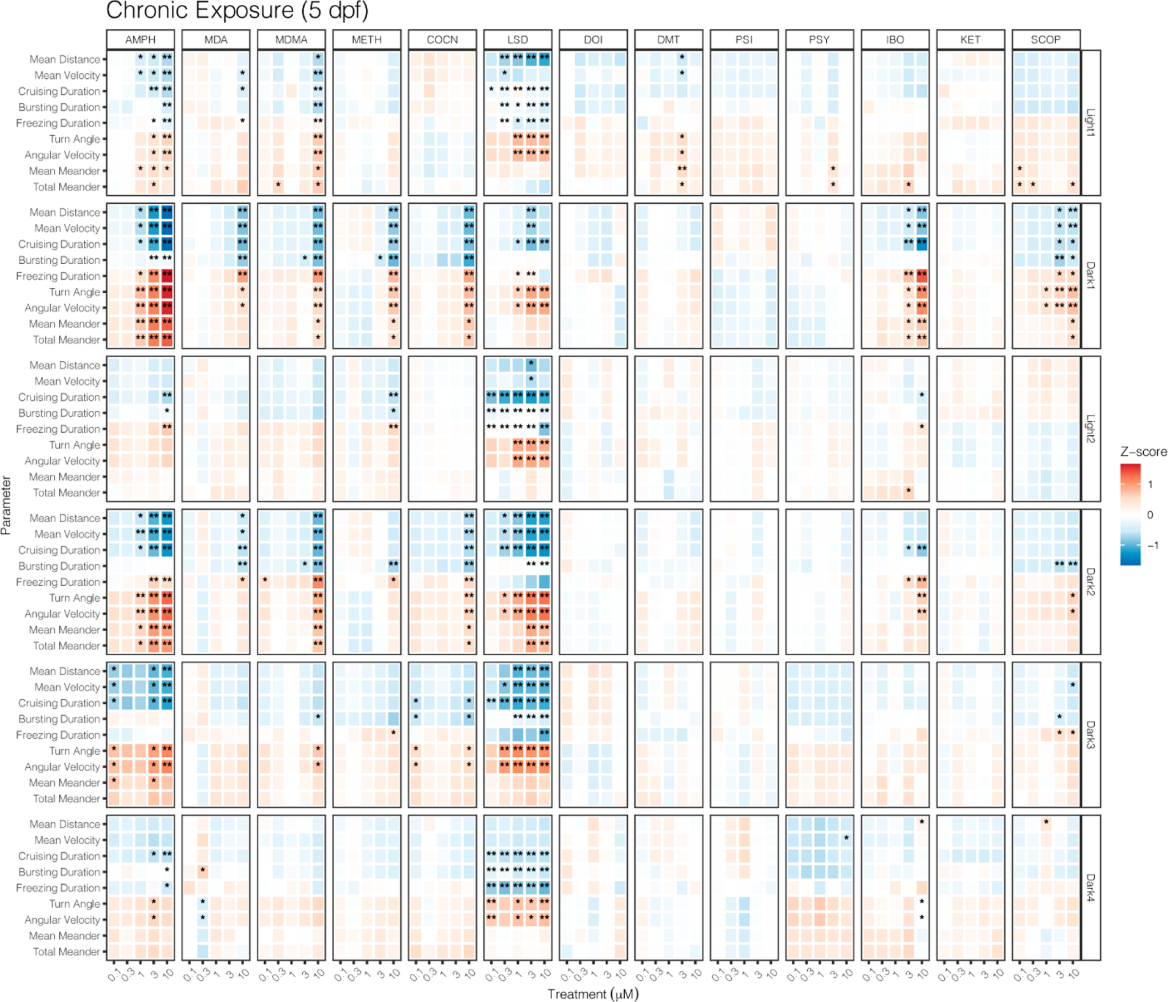
Z-score heatmap and statistical analysis of photomotor behavioral at 5 dpf after chronic developmental exposure.Top *x*-axis: compounds screened at 5 dpf after chronic developmental exposure. Bottom *x*-axis: concentrations of compounds. Left *y*-axis: tracked and analyzed parameters (see Table 2). Right *y*-axis: light cycles (see Figure 1). Z-score heatmap shading indicates increases (red shading) and decreases (blue shading) compared to control fish (Vehicle: 0.1% (v/v) DMSO). Asterisks (*, **) indicate significant differences (*p* < 0.05, *p* < 0.01, respectively) compared to control fish (*n* = 41–48 fish from three separate spawns).

By alternating light and dark phases during behavioral testing, we were able to assess the ability of the fish to sense changes in light stimuli and correspondingly alter their swimming behavior.^34^ Swimming behavior in response to alternating light and dark epochs (Figures 2 and 3) was analyzed during Light 1 (5 min), Dark 1 (5 min), Light 2 (5 min), and Dark 2–4 (5 min each) for a total of 30 min. The last three dark epochs are designed to test acclimation after an extended exposure to dark conditions. The Dark 2 epoch allowed for the analysis of photomotor behavioral responses induced by the switching from light to dark, whereas the Dark 3 and Dark 4 epochs that immediately followed without a change in light stimulation allowed us to observe the effects of chemical exposures on the typical habituation of the motor response to continued dark. Significant changes in swimming behavior compared to vehicle control fish suggest nervous system dysfunction.^35−37^

Generally, chemical-induced behavioral alterations observed at 4 dpf (Figure 2) were either similar to or less pronounced than those observed at 5 dpf (Figure 3). Interestingly, bursting duration appears to be a parameter that may differentiate psychostimulant compounds from hallucinogenic compounds since 10 μM COCN, MDA, MDMA, and METH decreased bursting duration at 4 dpf (Figure 2), while no significant changes were observed for LSD, DOI, DMT, PSI, PSY, IBO, KET, and SCOP. The only psychostimulant that did not follow this trend was AMPH, which did not evoke a significant change in bursting duration compared to the vehicle control at 4 dpf (Figure 2).

Anxiety-like behavior in zebrafish embryos is complex, but typically manifests as erratic movement, which can be observed as increases in freezing duration, turn angle, angular velocity, mean meander, and total meander compared to vehicle treated fish.^33^ Many of these parameters were increased compared to the vehicle control by exposure to the psychostimulants AMPH, MDA, MDMA, METH, and COCN as well as the hallucinogenic compounds LSD, IBO, and SCOP in a number of the light/dark epochs (right *y*-axes in Figures 2 and 3), but differences in the parameters were most pronounced in Dark 1. Swimming parameters for a given condition in a particular epoch that were statistically different from the vehicle control fish are indicated with asterisks and the directional change is depicted as a z-score heatmap (Figures 2 and 3). Another similarity between these eight chemicals is that they all reduced overall movement observed in Dark 1 (Figures 2 and 3) as shown by decreases in mean distance, mean velocity, cruising duration, and bursting duration, suggesting that these compounds produce sedative or akinesia phenotypes. This type of generalized sedative response in zebrafish is a common response to compounds that induce dopamine depletion.^33^

While AMPH, MDA, MDMA, METH, COCN, LSD, IBO, and SCOP impacted several behavioral parameters in more than one light cycle, these phenotypes were strongest and present across the widest array of chemical exposures during the Dark 1 epoch at 5 dpf (Figure 3). LSD evoked a particularly strong phenotype that was present in every epoch tested at both 4 and 5 dpf. Unlike psychostimulants and LSD, KET, tryptamine psychedelics (i.e., DMT, PSI, and PSY), and the amphetamine psychedelic DOI produced minimal observable behavioral phenotypes following chronic developmental exposure.

### Acute Exposure – Behavioral Assessment

**Figure 4:**
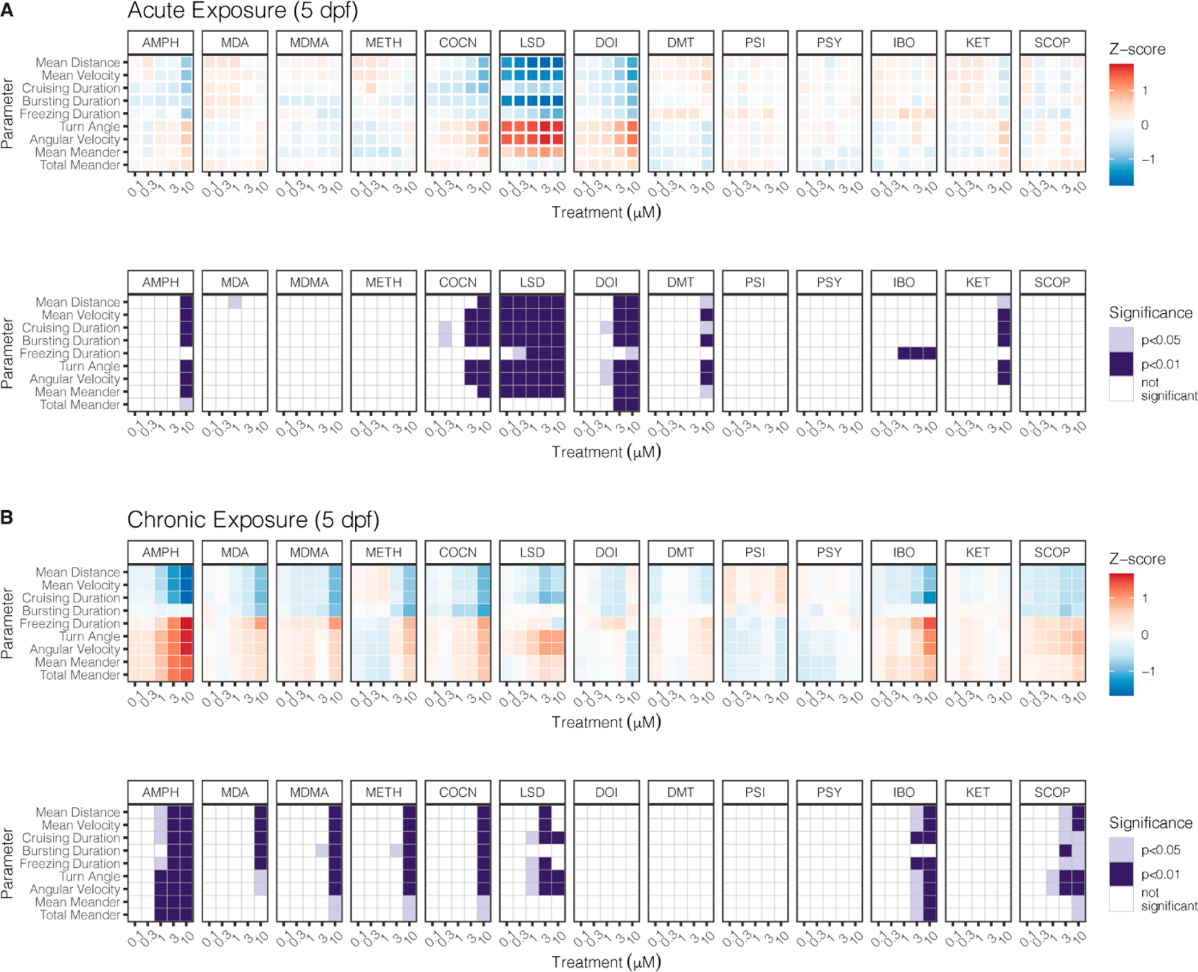
Acute vs chronic developmental exposures. (A) Z-score heatmap and statistical analysis of photomotor behavioral assay after acute exposure at 5 dpf. *n* = 39–48 fish from three separate spawns. (B) Z-score heatmap and statistical analysis of photomotor behavioral assay Dark 1 phase after chronic exposure at 5 dpf. *n* = 41–48 fish from three separate spawns.

The testing paradigm used following chronic exposures (Figure 1A) enabled observation of chemical effects on photomotor behavior. The testing paradigm used following acute exposures (Figure 1B) differed in that changes in light levels were eliminated, which allowed assessment of the acute effects of chemical exposure on motor responses and/or sedation. At 4 dpf, we transferred one fish per well to 96-well plates. At 5 dpf, we exposed the fish to light during a 10 min acclimation period prior to the addition of compounds, and behavior was then monitored for 30 min (Figure 1). Of the 13 compounds tested, five (AMPH, COCN, DOI, KET, and LSD) produced significant changes in multiple behavioral parameters following acute administration (Figure 4A). Chronic exposure to COCN and LSD (Figure 4B) produced similar effects to acute exposures (Figure 4A), leaving open the possibility that the responses observed following chronic exposure result from the acute effects of the compounds. Acute and chronic exposure to AMPH also produced similar behavioral effects; however, the magnitude of the effects was quite different. Chronic administration of AMPH produced a much stronger phenotype than acute treatment, suggesting that it causes developmental toxicity.

While chronic exposure to DOI and KET did not induce behavioral alterations compared to vehicle control fish (Figure 4B), these compounds caused significant differences in our acute exposure paradigm (Figure 4A). These results may reflect rapid metabolism or acclimation to these compounds by the fish during the chronic exposures. The robust responses in nearly all parameters analyzed for the 3 μM and 10 μM DOI acute exposure groups (Figure 4A) mimicked those of chronic exposures to AMPH, MDA, MDMA, METH, COCN, LSD, IBO, and SCOP (Figures 2 and 3), demonstrating a phenotypic link between these compounds. This may be due to the indirect effects of DOI on dopamine levels as demonstrated in previous studies.^38,39^

Acute exposure to MDA, MDMA, METH, and SCOP did not show significant differences (*p* < 0.01) compared to vehicle control fish (Figure 4A), indicating that chronic exposure to these compounds caused behavioral deficits by altering development (Figure 4B). It is worth noting that the psychostimulants tested in our acute studies (AMPH, MDA, MDMA, METH, and COCN) induced similar behavioral responses after chronic administration, and the effects of acute exposure to AMPH and COCN as well as chronic exposure to MDMA on mean distance traveled are consistent with previous studies.^40,41^ Currently, it is unclear why AMPH produces more pronounced neurotoxicity compared to other psychostimulants, but these results are important given that a large number of children are prescribed AMPH for treating attention-deficit/hyperactivity disorder.

While most chronically administered compounds produced similar behavioral effects at 4 and 5 dpf, the effects of scopolamine were more pronounced at 5 dpf. This underscores the importance of investigating and testing drugs on more than 1 day during development. Developmental processes are complex, with many changes occurring over a short period, so analyzing the effects of various exposures at multiple time points can uncover additional phenotypes in larval zebrafish. Given that SCOP has been shown to readily cross the placenta,^42−44^ our studies reveal potential risks associated with administering SCOP to treat nausea during pregnancy.^45^

Like chronic exposure, acute exposure to the hallucinogenic tryptamines, DMT, PSI, and PSY, did not significantly alter photomotor behavior compared to vehicle control fish (Figure 4). These compounds are believed to act primarily through activation of serotonin receptors such as the 5-HT2A receptor. In contrast, the other hallucinogenic compounds (LSD, DOI, KET, IBO, and SCOP) tested in our assays have been shown to either directly or indirectly impact dopamine signaling.^38,39^ The lack of phenotypic differences in either acute or chronic exposure paradigms could reflect the fact that DMT, PSI, and PSY are rather selective serotonergic psychedelics or that they are rapidly metabolized and cleared from the body.

## Conclusions

Using larval zebrafish, we demonstrated that chronic and/or acute exposure to several psychostimulants and hallucinogens can cause adverse developmental behavioral outcomes in the absence of overt teratological effects. Surprisingly, we found that zebrafish were less sensitive to hallucinogenic psychoplastogens like KET, DMT, PSI, and PSY than we had originally hypothesized. In stark contrast, compounds that modulate dopaminergic signaling produced significant neurodevelopmental toxicity. Our work underscores the usefulness of zebrafish models for directly comparing the neurotoxicity profiles of related compounds and further delineates the risks associated with exposure to psychostimulants and some hallucinogens during neurodevelopment.

## 3 Materials and Methods

### Chemicals

All chemicals used in this study along with their chemical and common names and classifications are listed in Table 1. The NIH Drug Supply Program provided the following compounds: (+)-lysergic acid diethylamide hemitartrate (LSD, CAS: 17676-08-3), psilocin (PSI, CAS: 520-53-6), psilocybin (PSY, CAS: 520-52-5), (−)-ibogaine hydrochloride (IBO, CAS: 36415-61-9), and (−)-cocaine hydrochloride (COCN, CAS: 53-21-4). The following compounds were purchased from commercial sources: (±)-ketamine hydrochloride (KET, Fagron, 803647, CAS: 1867-66-9), (±)-2,5-dimethoxy-4-iodoamphetamine hydrochloride (DOI, Cayman, 13885, CAS: 42203-78-1), (±)-methylenedioxymethamphetamine hydrochloride (MDMA, Cayman, 13971, CAS: 64057-70-1), and (−)-scopolamine hydrobromide trihydrate (SCOP, Acros Organics, AC161750010, CAS: 6533-68-2). The remaining compounds used in these studies were synthesized in house and judged to be of >95% purity based on nuclear magnetic resonance (NMR) and ultrahigh performance liquid chromatography–mass spectrometry (UHPLC). (±)-Amphetamine fumarate (AMPH) and (±)-3,4-methylenedioxyamphetamine fumarate (MDA) were prepared using methodology adapted from Nenajdenko et al.^46^ (±)-Methamphetamine fumarate (METH) was prepared as a 1:1 ratio of the enantiopure *R*- and *S*-methamphetamine fumarate synthesized as previously described.^47^ The vehicle used for all compounds was molecular biology grade dimethyl sulfoxide (DMSO, ACROS, AC327182500, CAS: 67-68-5).

### Zebrafish Husbandry

All zebrafish work was approved and performed in accordance with the University of California Davis Institutional Animal Care and Use Committee (IACUC). Adult wildtype zebrafish (5D) were initially obtained from the Sinnhuber Aquatic Research Laboratory (SARL) at Oregon State University (Corvallis, Oregon), and subsequent generations were raised at UC Davis. Adult zebrafish were maintained under controlled conditions consisting of a 14:10 h light (∼850 lx):dark photoperiod, water temperature of 28.5 ± 0.5 °C, pH of 7.2 ± 0.4, and conductivity of 700 ± 100 μS. Adult fish were fed twice daily with commercial flake foods GEMMA Micro (Skretting, Tooele, Utah) and spawned naturally in groups of 4–6 fish. Embryos were collected and staged following fertilization as previously described.^48^ Embryos were kept in an incubator at 28.5 °C until plated for chemical exposures.

### Chemical Exposures

For chronic developmental exposures (Figures 2, 3, and 4B), zebrafish embryos were obtained from natural group spawning and age-matched within the first hour of fertilization. Enzymatic dechorionation took place at 4 h postfertilization (hpf), where ∼800 embryos were placed in a round glass plate (10 cm diameter) with 25 mL system water (same water in which fish were raised) to which 50 μL of 63.6 mg/mL (∼11.12 U) Pronase E (protease from *Streptomyces griseus*, ≥ 3.5 U/mg, P5147 Sigma-Aldrich, St. Louis, Missouri) was added. Constant manual plate agitation took place for 6 min following a wash with 2 L of system water.^49^ Dechorionated embryos were randomly placed in individual wells in 96-well plates (Falcon, Fisher Scientific, Hampton, New Hampshire) with 100 μL embryo medium (EM).^50^ Chemical stocks (1000×) were diluted to 2× in EM, and 100 μL was added to each well, resulting in final concentrations 0.1, 0.3, 1, 3, and 10 μM in 0.1% DMSO. Each experimental group included 16 fish per spawning from three separate spawning events, resulting in a total of *n* = 48 per group. Plates were covered in Parafilm M (Bemis, North America, Neenah, Wisconsin) and placed in a 28.5 °C light-controlled (14 h light (∼300 lx):10 h dark) incubator for static waterborne exposure through 5 dpf.

For acute developmental exposures (Figure 4A), embryos were also obtained by natural group spawning. Embryos were raised in Petri dishes without chemically induced dechorionation until 4 dpf, when they were transferred to 96-well plates with 100 μL EM. At 5 dpf, exposures were conducted identically to chronic developmental exposures (100 μL of compounds at 2× of the final concentration was added to each well) with the exception that behavioral assessments were conducted immediately after addition of chemical compounds to wells containing zebrafish larvae.

### Teratological Assessment

Fish were examined by investigators blinded to experimental group every day of the exposure period using a stereoscope (Olympus Stereo Microscope Model SZ61, Olympus, Japan) with a maximal magnification of 4.5×. On 4 and 5 dpf, fish were examined for teratological effects immediately after behavioral assessment. Individual fish were scored for mortality, signified by an absence of heartbeat or structural disintegrations of the fish, and gross morphological abnormalities including those of the caudal fin, pectoral fin, eyes, and jaw, as well as arrested development, abnormal axis, and edemas in the pericardium and yolk sac. Scores were called in a binary fashion, i.e. a malformation was or was not observed. Percent incidence of total dead, malformed, and viable fish was calculated for each experimental group on each day (Figure S1).

### Photomotor Behavior

After removing the plate lid and Parafilm, 96-well plates with exposed fish were placed into the Noldus behavioral chamber (Noldus, Netherlands). Fish developmentally exposed to chemical compounds were acclimated in the light (∼1900 lx) for 10 min, then subjected to a series of 5 min light (∼1900 lx) and dark (∼0 lx) epochs for a total of 30 min: Light 1 (5 min), Dark 1 (5 min), Light 2 (5 min), Dark 2 (5 min), Dark 3 (5 min), and Dark 4 (5 min). Following acute exposures, larvae were subjected to one 30 min light (∼1900 lx) epoch. Fish were recorded using an infrared GigE camera (Noldus) at 30 frames per second and motor behavior was analyzed using the EthoVisionXT software (Noldus). Locomotor parameters were extracted from the software into Excel (Microsoft, Albuquerque, New Mexico) spreadsheets.

### Behavioral and Statistical Analyses

Data were collected from three separate spawns to ensure genetic variability and address interspawn differences. We analyzed behavioral differences in the swimming parameters defined in Table 2 with significant differences (*p*-values <0.05 and <0.01) compared to vehicle control fish. Averages for each locomotor parameter were collected and exported from EthoVision into an Excel file (.xlsx) and converted to .csv files. All further data processing and statistical analysis were conducted in R version 4.1.1.^51^ Statistical analyses were conducted using previously described methods.^52^ Data were tested for normality and homogeneity of variance using Shapiro Wilkes and Levene’s tests, respectively, using the package rstatix.^53^ Nonparametric Kruskal–Wallis Analysis of Variance tests were conducted using the rstatix package to determine whether differences existed across groups. Posthoc analyses were performed to identify differences between vehicle controls and exposed groups using multiple comparison tests at alpha <0.05 using the emmeans package.^54^ The *p*-values were adjusted using Dunnett’s *p*-value adjustment method in the emmeans package. For presentation purposes, a Z-score was calculated within each experimental group across parameters and normalized to vehicle control larvae to visualize the directional change of each behavioral parameter on the same scale. Z-scores were calculated in R using the equation: Z = (x – μ)/σ, where within a condition, x is the observed value, μ is the mean, and σ is the standard deviation. Code for statistical analysis and graphing along with example data can be found at (https://github.com/insideafish).

## Supporting Information Available

Teratological data and chemical synthesis data (PDF)
