# Supplementary material for: Developmental Neurotoxicity Screen of Psychedelics and Other Drugs of Abuse in Larval Zebrafish (Danio rerio)
Source: ACS Chem Neurosci. 2023 Feb 8;14(5):875–84. doi: 10.1021/acschemneuro.2c00642 (PMC9983010; doi:10.1021/acschemneuro.2c00642)
Supplement: Supplementary file 1 — cn2c00642_si_001.docx [file cn2c00642_si_001.docx]

**Developmental Neurotoxicity Screen of Psychedelics and Other Drugs of Abuse in Larval Zebrafish (*Danio rerio*)**

**Supporting Information**

*(5 pages)*

Robert J. Tombari^1^ , Paige C. Mundy^2^, Kelly M. Morales^2^, Lee E. Dunlap^1^, David E. Olson,^1,3,4,5*^ and Pamela J. Lein^2,4*^

* Correspondence: [deolson@ucdavis.edu](mailto:deolson@ucdavis.edu); pjlein@ucdavis.edu

**Affiliations:**

^1^ Department of Chemistry, University of California, Davis, Davis, CA 95616, USA

^2^ Department of Molecular Biosciences, University of California, Davis, Davis, CA 95616, USA

^3^ Department of Biochemistry & Molecular Medicine, School of Medicine, University of California, Davis, Sacramento, CA 95817, USA

^4^ Center for Neuroscience, University of California, Davis, Davis, CA 95618, USA

^5^ Institute for Psychedelics and Neurotherapeutics, University of California, Davis, Davis, CA 95616, USA

***Contents***

*Figure S1*

*1. Experimental Procedure for the Synthesis of MDA Fumarate*

*2. Experimental Procedure for the Synthesis of Amphetamine Fumarate*

**
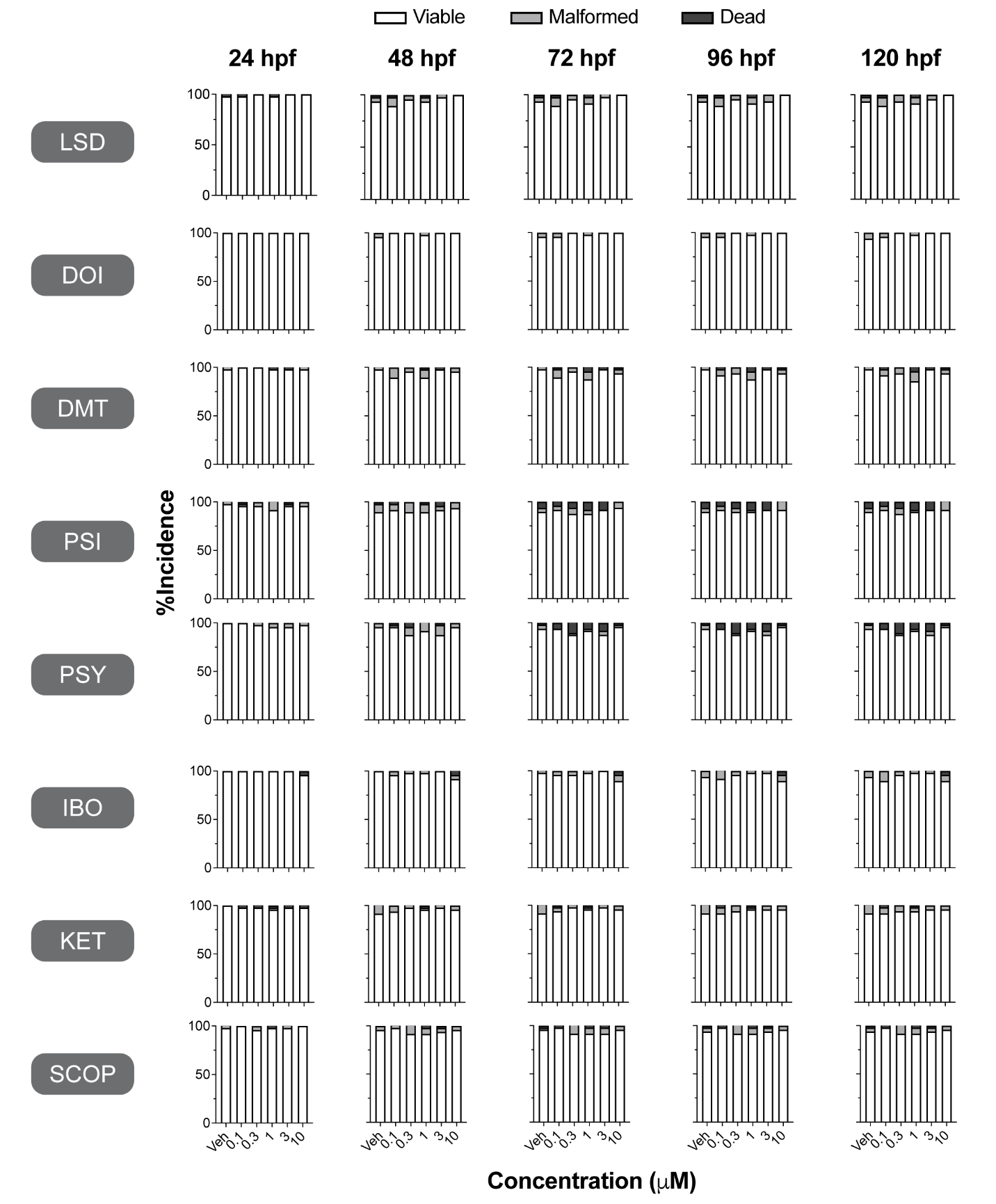
**


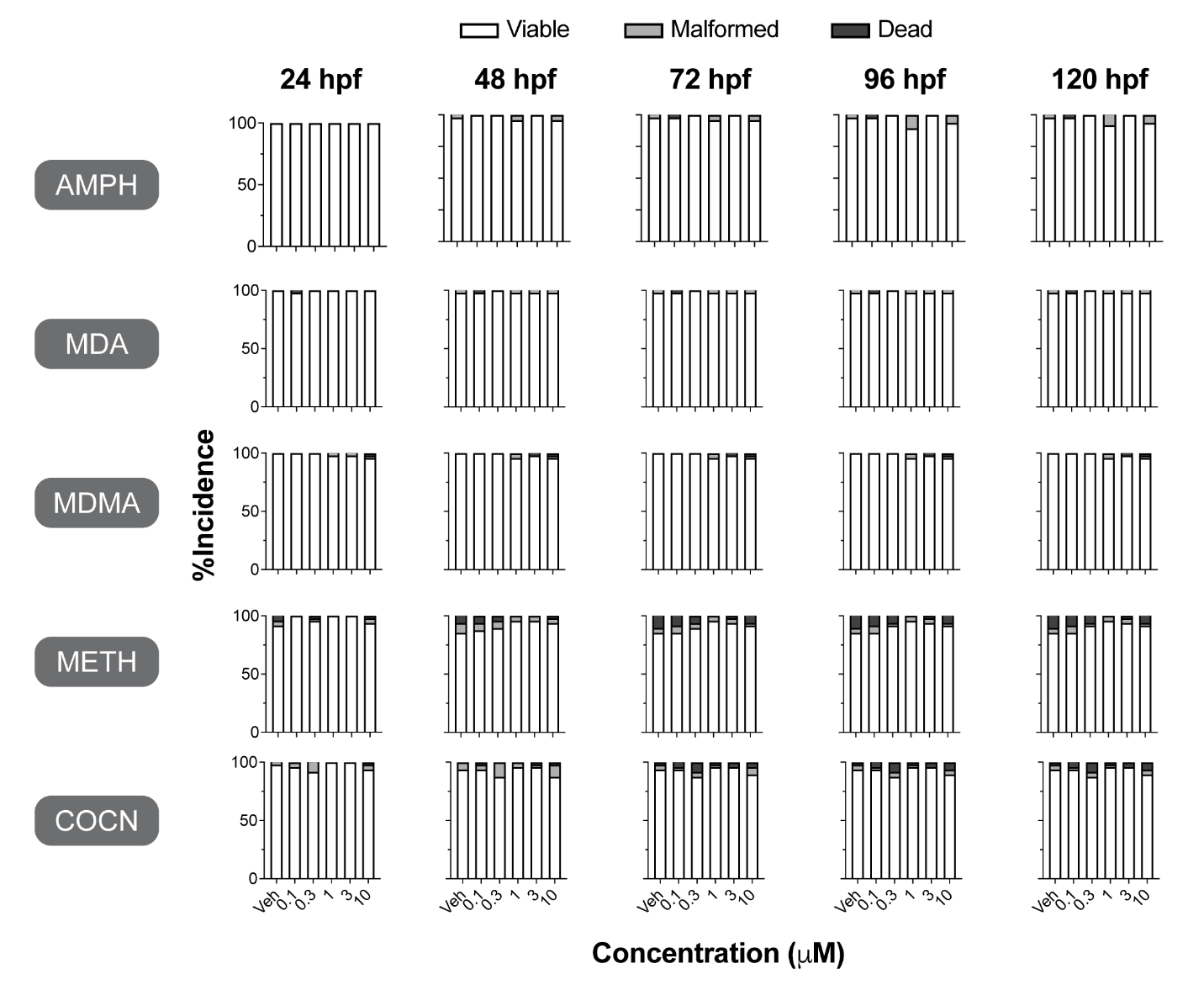


**Figure S1. Several common drugs of abuse do not produce any morphological or teratogenic effects in developing zebrafish.** Teratological and mortality assessment of fish after 1, 2, 3, 4, and 5 dpf of exposure to vehicle (Veh) or compounds (0.1, 0.3, 1, 3, or 10 μM). Viable (white bar), malformed (gray bar), dead (black bar) fish represented as percent incidence (n = 48 fish from three separate spawns).

1. **Synthesis of MDA Fumarate.**

*5-(2-nitroprop-1-en-1-yl)benzo[d][1,3]dioxole*. To a solution of piperonal (1.0 g, 6.6 mmol) in nitroethane (4 mL) was added ammonium acetate (0.26 g, 3.3 mmol, 0.5 equiv). The reaction was refluxed overnight. The reaction mixture was cooled to -20 °C and the wall of the flask was scraped with a glass rod to initiate crystallization. The crystals were filtered and washed with cold ethanol. The resulting yellow solid was dried under reduced pressure to yield the desired compound. Yield = 0.716 g, 52%.

*MDA Fumarate.* To an ice-cold solution of 5-(2-nitroprop-1-en-1-yl)benzo[d][1,3]dioxole (0.76 g, 2.1 mmol) in THF (23 mL) was added lithium aluminum hydride (4M in THF) (1.7 mL, 7.0 mmol, 2. equiv) dropwise. The reaction was heated at 66 °C overnight before being cooled to -10 °C and diluted with THF (3mL) followed by addition of a saturated solution of Rochelle’s salt (5 mL). The resulting solution was diluted with EtOAc (30 mL) and a saturated solution of Rochelle’s salt (75 mL). The phases were separated, and the aqueous phase was extracted with EtOAc (2 × 30 mL). The organic extracts were combined, dried over Na_2_SO_4_, filtered, and concentrated under reduced pressure. The unpurified material was dissolved in CHCl_3_ (1 mL) and was added to a boiling solution of fumaric acid (0.21 g, 1.8 mmol, 0.5 equiv) in THF (20 mL). A precipitate formed immediately, and the solution was cooled to -20 °C before being filtered and washed with cold THF. The resulting white solid was dried under reduced pressure to yield the pure compound as the fumarate salt (1:1). Yield = 0.50 g, 49%. ^1^H NMR (400 MHz, CD_3_OD) δ 6.78 (m, 2H), 6.69 (m, 3H), 5.93 (s, 2H), 3.46 (m, 1H), 2.87 (dd, *J* = 13.8, 6.6 Hz, 1H), 2.73 (dd, *J* = 13.8, 7.8 Hz. 1H), 1.25 (d, J = 6.59 Hz, 3H,) ppm; ^13^C NMR (100 MHz, CD_3_OD) δ 171.56, 149.58, 148.37, 136.30, 130.97, 123.55, 110.39, 109.47, 102.46, 50.27, 41.51, 18.33 ppm. LRMS (ES+) m/z calcd for C_10_H_13_NO_2_+ 179.09, found 180.27 (MH+).

1. **Synthesis of Amphetamine Fumarate.**

*(2-nitroprop-1-en-1-yl)benzene.* To a solution of benzaldehyde (4 mL, 39 mmol) in ethanol (4 mL) was added nitroethane (2.8 mL, 47 mmol, 1.2 equiv.) and butyl amine (0.20 mL, 3.9 mmol, 0.1 equiv). The reaction was heated with stirring at 78 °C overnight. The reaction mixture was cooled to -20 °C and the wall of the flask was scraped with a glass rod to initiate crystallization. The crystals were filtered and washed with cold ethanol. The resulting yellow solid was dried under reduced pressure to yield the desired compound. Yield = 3.9 g, 61%.

*Amphetamine Fumarate.* To an ice cold solution of (2-nitroprop-1-en-1-yl)benzene (2.2 g, 13.6 mmol) in THF (82 mL) was added lithium aluminum hydride (6.5g, 173 mmol, 12 equiv). The reaction was refluxed overnight before being cooled to -10 °C and diluted with IPA (7 mL), 1M NaOH (7mL), and H_2_O (14mL) and stirred for 30 min. The reaction mixture was filter and the filtrate was acidified with 1M HCl and extracted with DCM (3 × 50mL). The Aqueous layer was basified with 1M NaOH and extracted with DCM (3 × 50 mL). The organic extracts were combined, dried over Na_2_SO_4_, filtered, and concentrated under reduced pressure. The unpurified material was dissolved in acetone (5 mL) and was added to a boiling solution of fumaric acid (1.6 g, 13.6 mmol, 1 equiv) in acetone (200 mL). A precipitate formed immediately, and the solution was cooled to -20 °C before being filtered, washing with cold acetone. The resulting white solid was dried under reduced pressure to yield the pure compound as the fumarate salt (1:1). Yield = 2.3 g, 47%. ^1^H NMR (400 MHz, CD_3_OD) δ 7.35 (d, *J* = 7.3 Hz, 2H), 7.26 (m, 3H), 6.69 (s, 2H), 3.51 (m, 1H), 3.01 (dd, *J* = 13.1, 5.8 Hz, 1H), 2.79 (dd, *J* = 13.1, 8.2 Hz, 1H), 1.25 (d, *J* = 6.7 Hz, 3H,) ppm; ^13^C NMR (100 MHz, CD_3_OD) δ 171.61, 137.47, 136.30, 130.36, 129.96, 128.33, 50.16, 41.83, 18.28 ppm. LRMS (ES+) m/z calcd for C_9_H_13_N_2_+ 135.10, found 136.26 (MH+).
